# The Role of Circular Frames in the Management of Tibial Fractures

**DOI:** 10.7759/cureus.3356

**Published:** 2018-09-25

**Authors:** Gerard A Sheridan, Kevin Clesham, S.R. Kearns

**Affiliations:** 1 Orthopaedics, University Hospital Galway, Galway, IRL

**Keywords:** tibia, fracture, circular frame, bone graft, infection

## Abstract

Introduction

The purpose of this study is to evaluate the factors that impact the clinical and radiographic outcomes in a patient cohort undergoing external fixation for tibial fractures. We also aim to investigate the use of autograft, allograft, and synthetic bone graft in varying combinations in the setting of tibial bone loss with respect to optimal times to union.

Methods

This was a retrospective study, including 46 patients treated with a circular external fixator for the management of acute tibial fractures. The study was carried out over a 10-year period, between 2007 and 2017, at our institution. The mean follow-up at the time of review was 4.6 years. Primary outcome measures were ‘time to union,’ ‘delayed union’ (> 6 months), ‘infection,’ and ‘duration of external fixation.’ Secondary outcomes included 'length of hospital stay' and functional scores using the 'Short Form-12 (SF-12).' The statistical analysis included both univariate and multivariate analyses to control for confounding variables when assessing predictors of delayed union and infection.

Results

Forty-six patients fulfilled the study criteria. Fifteen fractures were classified as open. The mean number of procedures per patient was 3.8 and the mean length of stay per patient was 33 days. The mean time to union was 8.6 months overall. Significant predictors of prolonged time to union were the ‘number of interventions’ (p<0.01) the patient underwent and the ‘bone graft type’ (p<0.01) used. The time to union in the presence of either autograft or allograft was lengthened by the addition of synthetic graft. Five patients developed a deep tissue infection. The use of synthetic bone graft was significantly associated with infection (p<0.05). On subgroup analysis, it was found that the use of synthetic graft in any combination leads to significantly higher rates of deep tissue infection (p<0.05). The mean time to full weight bearing was 10.6 months (s=9.78, 3-36). The majority (57%) were using walking aids and 67% reported that the injury was still affecting their lifestyle.

Conclusion

Tibial fractures with bone loss are a complex group of injuries that often require multiple surgical interventions, prolonged hospital inpatient stay, and suboptimal functional outcomes in many cases. The best times to union are achieved when autograft is used alone without any other combination of bone graft type. The use of synthetic bone graft also significantly increases the rate of deep tissue infection in this cohort. We recommend the use of autograft alone when treating bone defects in tibial fractures with external fixators.

## Introduction

The purpose of this pilot study is to evaluate the factors that impact the clinical and radiographic outcomes in a patient cohort undergoing external fixation for tibial fractures. Specifically, we note that the optimal bone graft type for managing bone loss in tibial fractures has not been clearly defined in the literature to date. We aim to evaluate the most effective combination of bone graft types available to treat these fractures in relation to union rates, time to union, and infection.

## Materials and methods

This was a retrospective study including patients treated with a circular external fixator for the management of acute tibial fractures. Patients treated between 2007 and 2017 at our University Hospital under a single foot and ankle specialist surgeon were eligible for inclusion. Exclusion criteria included incomplete clinical follow-up and external fixator application for any indication other than acute tibial fracture.

Theater implant records were used to identify all patients. Clinical records, radiographic images, laboratory results, and patient-reported outcome measures (Short Form-12 (SF-12)) from the outpatient setting were all collected by the researchers. Patients were followed from presentation through to union and removal of external fixation.

The clinical course of each patient involved initial stabilization of the fracture in the emergency department. Appropriate antimicrobial prophylaxis was instigated immediately and open wounds were treated as appropriate. Every patient had a circular external fixator applied initially and were definitively managed in this device. Some patients underwent additional open reduction internal fixation (ORIF) in conjunction with the external fixator. A mixture of autograft, allograft, and synthetic bone graft (recombinant human bone morphogenic protein seven (rhBMP-7) (eptotermin alfa)) was used, depending on the degree of bone loss. The iliac crest was the autograft donor site of choice and the femoral head was the allograft used. In cases of deep tissue infection, the two-stage 'induced-membrane’ technique was used [1].

Independent and dependent variable values were recorded. Primary outcome measures were ‘time to union,’ ‘delayed union’ (> six months), ‘infection,’ and ‘duration of external fixation.’ The statistical analysis performed was determined by the nature of the variables involved. For categorical dependent and independent variables, the Chi-squared analysis was used provided there were five patients per group. The Fisher-Exact test was used in other cases. For interval-dependent variables, the Wilcoxon-Mann-Whitney test was used. A multivariate analysis was then used to control for confounding variables when assessing an outcome variable with multiple predictor variables. All statistical analysis was performed using Stata software (Stata/IC 13.1 for Mac; StataCorp, Texas, US (64-bit Intel; California, US)). A p-value of less than 0.05 was considered to be significant. Ethical approval was received before the commencement of this study.

## Results

Descriptive

Forty-six patients fulfilled the inclusion criteria. The mean age was 45 years with a 77% male preponderance. The mean follow-up was 4.6 years per patient (1-10, SD=2.76). Three patients were lost to follow-up and one patient opted for treatment in another facility. This left 42 patients with complete follow-up data.

**Figure 1:**
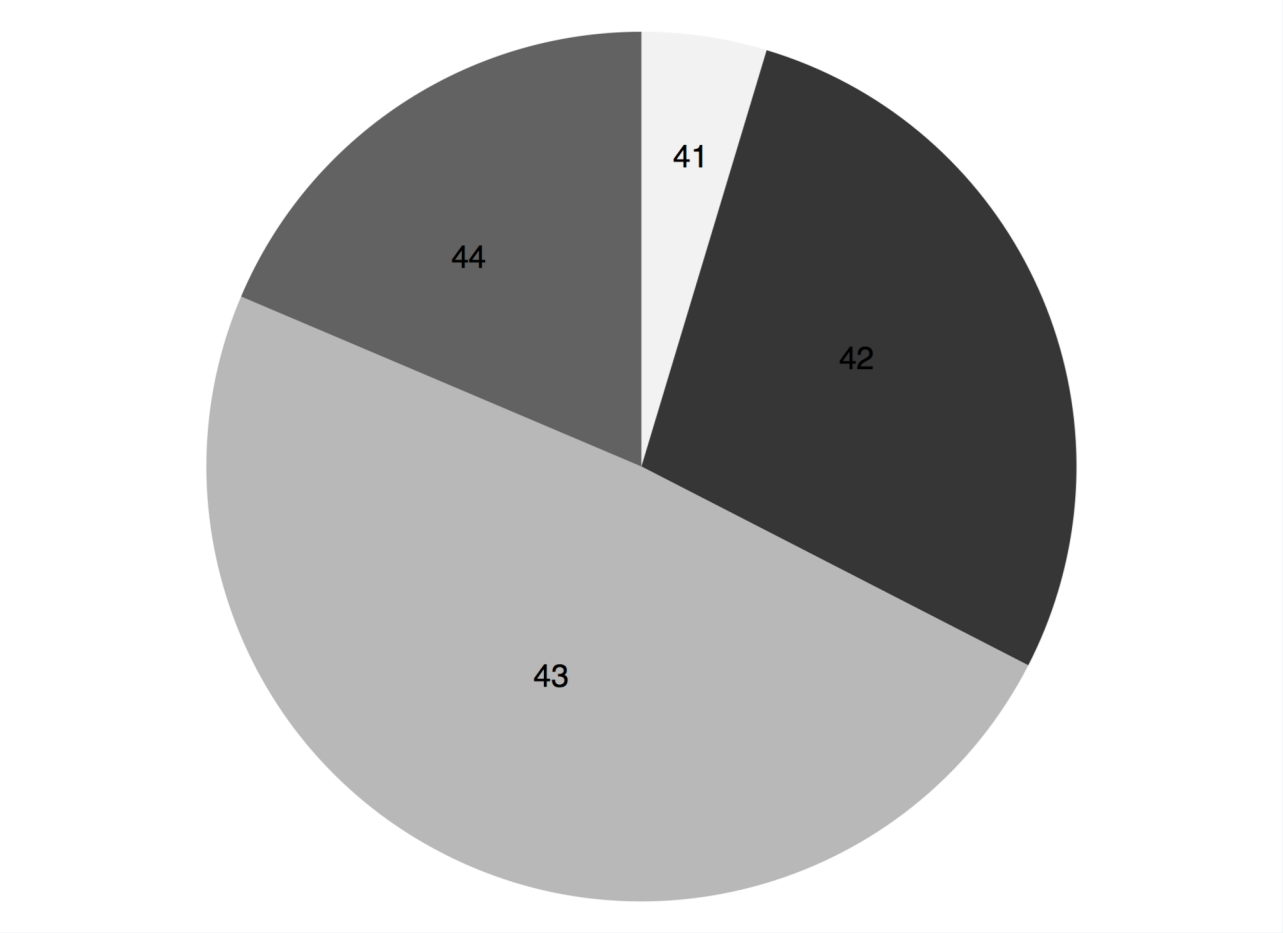
AO classification of tibial fractures AO: Arbeitsgemeinschaft für Osteosynthesefragen

One-third (15) of the fractures were classified as ‘open’ on presentation. According to the Arbeitsgemeinschaft für Osteosynthesefragen (AO) classification for tibial fractures, 16 patients were type 43-C (Figure 1) [2]. The commonest fracture type was a highly comminuted distal tibial intraarticular fracture.

The mean number of interventions per patient was 3.8 (SD=1.17, 2-6). The mean number of admissions was 3.5 (SD=1.73, 1-7) per patient. The mean length of stay (LOS) was 33 days (SD=32.5, 1-156) per patient with one patient requiring up to 156 days of admission. Seventeen patients (36%) underwent ORIF in conjunction with external fixation. Patients spent 6.3 months (SD=5.37, 1-30) on average in the external fixator. Those who underwent ORIF spent a mean of three months less in the external fixator (7.7 months vs 4.1 months). Fourteen (30%) patients underwent bone grafting at some point in their clinical course. Six patients received autograft alone while eight patients received synthetic bone graft in combination with either autograft or allograft.

Union

**Figure 2:**
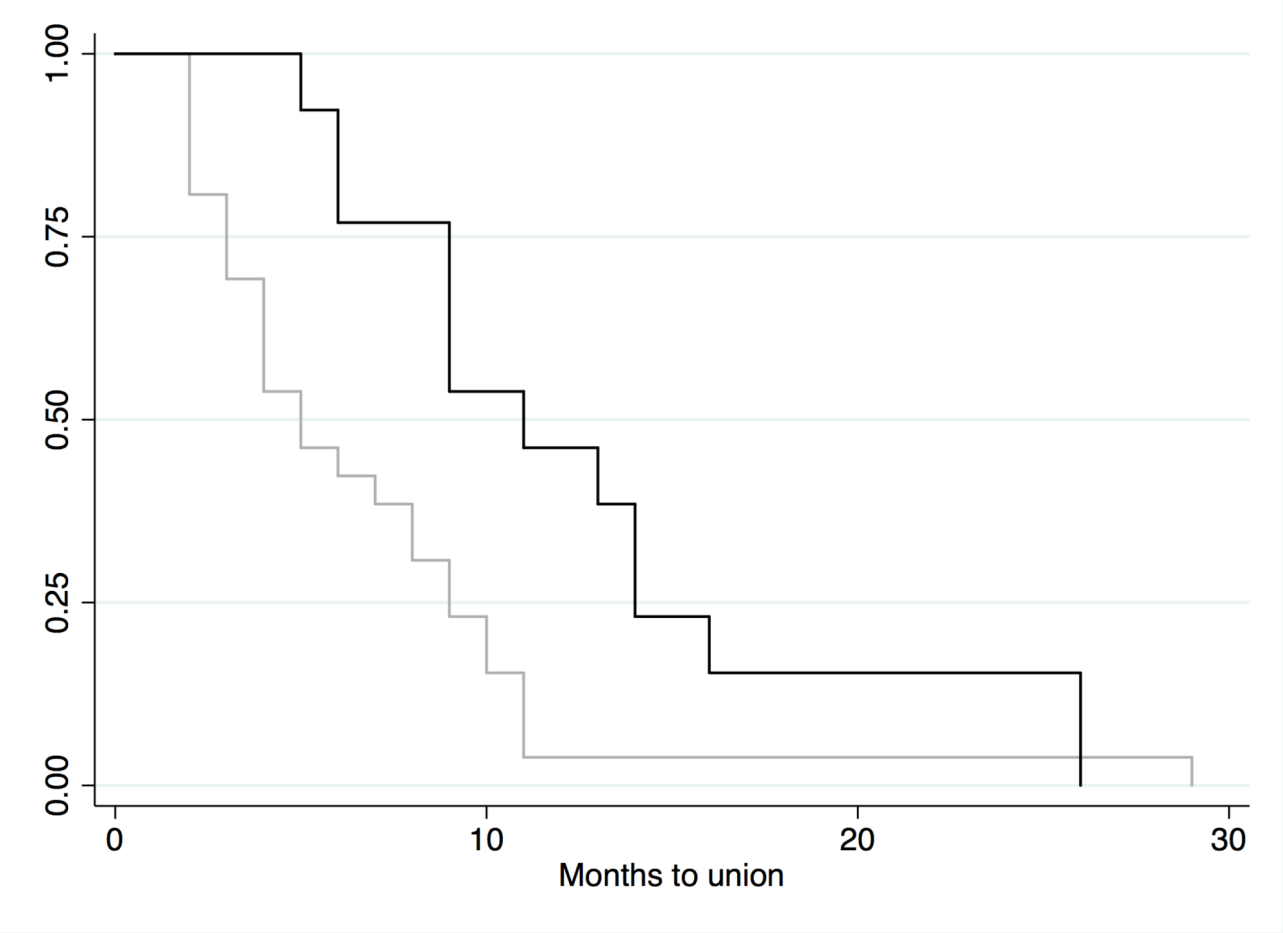
Kaplan-Meier curve, graft vs. no graft Black line=graft used (n=14); gray line=no graft used (n=32)

All fractures progressed to union. The mean time to union was 8.6 months (SD=6.57, 2-29 months). Fifty-three percent had delayed union. A univariate analysis illustrated that the predictors of a prolonged time to union were the ‘number of interventions’ (p<0.01) the patient underwent and the ‘bone graft type’ (p<0.01) used. Kaplan-Meier curves demonstrate the negative effect of bone graft usage on the time to union (Figure 2). The mean time to union in patients receiving autograft alone was 4.7 months. All other combinations included synthetic bone graft and in all cases, the mean time to union was greater than six months.

Seventeen patients developed an infection. If a deep infection was present, there was a 100% chance of delayed union compared to a 54% rate in the absence of deep infection.

A univariate analysis showed that the ‘number of interventions’ and the ‘graft type’ used had a statistically significant negative effect on ‘time to union.’ A multivariate analysis assessing ‘time to union’ was performed, controlling for the ‘number of interventions’ and the ‘graft type’ used. Analysis showed that the use of any bone graft significantly increased the time to union as compared with patients that received no bone graft at all (p<0.05).

Infection

Five patients developed a deep tissue infection. Another eight patients developed pin site infections and four developed superficial wound infections. Methicillin-sensitive Staphylococcus aureus (MSSA) was cultured in four of the five cases of osteomyelitis. Coagulase-negative staphylococcus (CNS) was cultured in isolation in one patient and in combination with MSSA in another patient.

**Figure 3:**
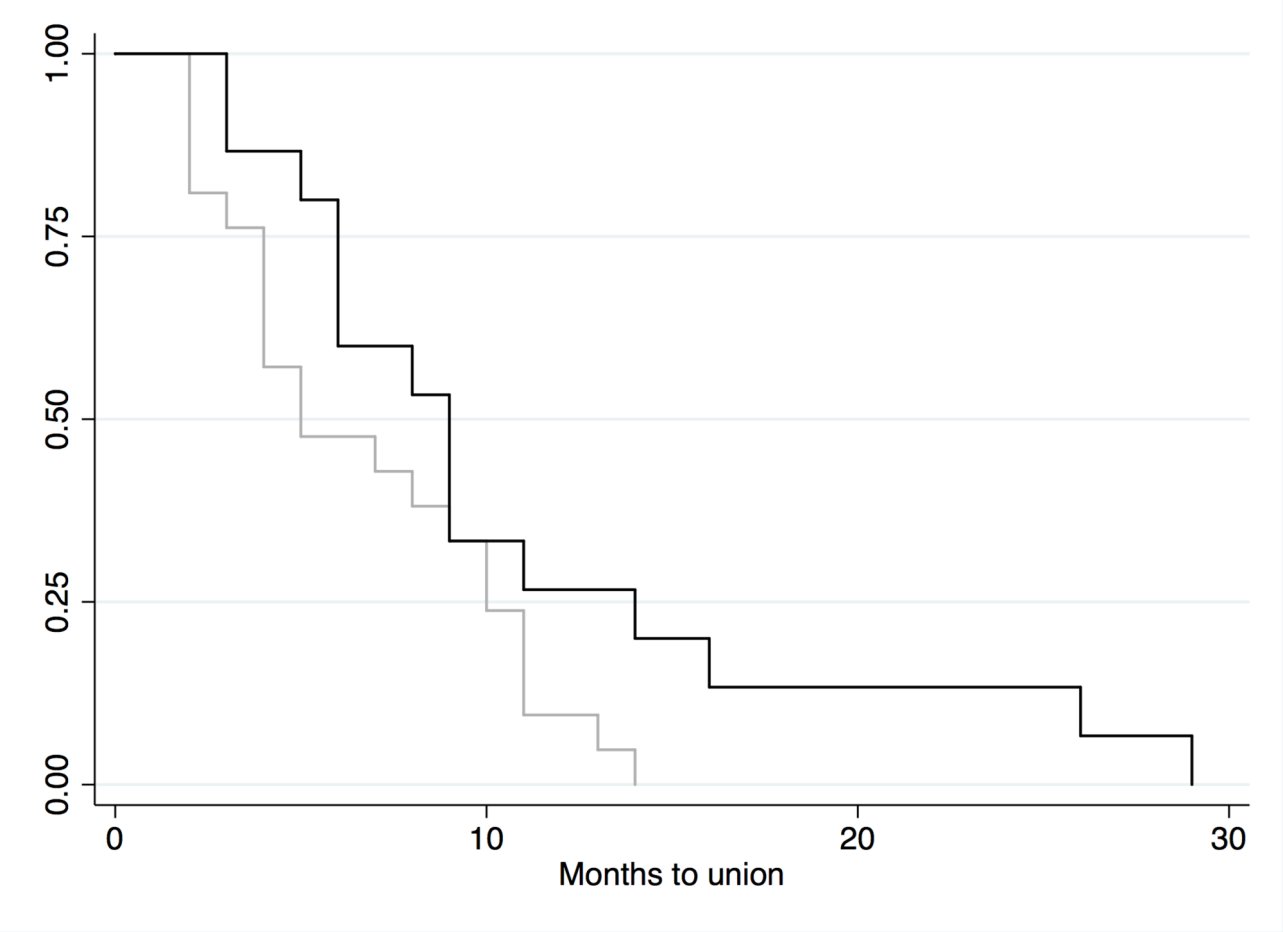
Kaplan-Meier curve, infection vs. no infection Black line=infection (n=17); gray line=no infection (n=29)

On univariate analysis, it was found that the use of synthetic bone graft in any combination leads to significantly higher rates of deep tissue infection (p<0.05). All infected cases had delayed union compared to only 54% of cases without deep infection. The following graph illustrates that there may be a trend, although not statistically significant, between infection and ‘time to union’ (Figure 3).

Lifestyle

The mean time to full weight-bearing (FWB) was 10.6 months (SD=9.78, 3-36). The majority of patients (57%) were using a walking aid and 67% said the injury was still affecting their lifestyle. SF-12 scores recorded showed a mean score of 26.9 (SD=3.52). Pre-injury scores were unavailable.

No independent variable could predict time to FWB or SF-12 scores. As discussed, the mean duration of the external fixator was just over six months. The use of bone graft and the type of bone graft used both predicted prolonged time in the external fixator (p<0.05). Interestingly, women spent less time in the external fixator overall (p<0.05). Smoking status had no impact on clinical outcomes.

## Discussion

The results of this study indicate that the use of bone graft, specifically synthetic bone graft, has a negative effect on time to union. Synthetic bone graft also significantly increases the rate of deep tissue infection. These are novel relationships, which have not been described in the literature to date.

Tibial fractures are complex injuries that have been treated with open reduction and internal fixation (ORIF) historically [3-4]. The use of external fixation is widely adopted in current practice to manage these very challenging fractures. The time to union between these two methods of fixation is comparable with reported higher rates of superficial infection and non-union with external fixation [5-6]. Recent evidence has also shown a comparable ankle range of motion, arthritis, and hindfoot scores between the two methods, confirming that both are reasonable management options in this injury type [7].

Regarding union rates, external fixation has been associated with very high union rates in the management of acute tibial fractures [5]. The use of open reduction and bone grafting in conjunction with external fixation has also been shown to impart very high union rates. Leung et al. describe 31 distal tibial fractures treated with these techniques, leading to union in all but one case [6]. Our study confirms union in all cases except for one patient who was still being actively treated for a delayed union at the time of review. Fifty-three percent of cases developed a delayed union, illustrating the challenge that faces orthopedic surgeons in managing fractures of this nature. Cases with deep infection had a 100% rate of delayed union compared to 54% of cases without a deep infection. Only five patients developed deep tissue infections. These figures are comparable to reported figures in the literature [6]. All deep tissue infections were treated with the Masquelet technique, with complete eradication of infection and union in all cases. Eight patients developed pin site infections. This is a well-described complication comparable to reported rates in the literature too [8].

The type of bone graft used and its impact on time to union has not been described in the literature to date. On the contrary, a study published by Ristiniemi in 2007 concluded that rhBMP-7 accelerated fracture healing and shortened the patients' sick leave [9]. This study looked at 20 patients who received rhBMP-7 who were matched with 20 patients who didn’t receive rhBMP-7. Unlike our study, Ristiniemi did not look at the numerous combinations in which bone graft can be and often is applied. Our subgroup analysis illustrates that the addition of rhBMP-7 to either autograft or allograft will lengthen the time to union. In fact, the only combination of graft that produced union in under six months was autograft alone.

In our study, 14 patients underwent bone grafting. Five patients received autograft alone. The mean time to union in these cases was 4.75 months in keeping with a normal union time. Two cases received autograft and synthetic graft with a mean union time of 7.5 months. Five patients receiving synthetic graft alone had delayed union times of 6.25 months. One case had a time to union of six months when synthetic graft was combined with allograft. Although these numbers are relatively small, the relationship is novel and seems to illustrate a negative impact of synthetic bone graft on time to union when compared to autograft alone. In short, rhBMP-7 usage prolongs the time to union when added to either autograft or allograft.

A synthetic graft also seems to play a negative role in the development of deep tissue infection in these patients. The use of bone graft in any combination only trends towards significance in this cohort. A subgroup analysis, however, demonstrates a significant relationship between the use of a synthetic graft and deep tissue infection. This is also a novel relationship not yet described in the literature.

These injuries also seem to have significant implications for the patients’ quality of life. We report that over half of our cohort was using a walking aid to mobilize at the time of review. Over 66% of patients felt that this injury was still affecting their lifestyle, despite the fact that the mean time of review after the injury was 4.6 years. Elsoe et al. found that only 27% of patients with tibial fractures had returned to employment 18 months after injury [10]. Volgas et al. reported that 37% of patients had to sell possessions to meet financial obligations after their injuries [11]. These figures illustrate the significant burden that patients endure as a result of these injuries.

There are limitations to this study. This is a retrospective study. Patients were not randomized to the type of bone graft they received. The bone graft was selected on a case-by-case basis. Confounding factors may also exist. There is a strong male preponderance, which may affect results. We have attempted to control for the majority of these factors using a multivariate analysis.

The sample size of 46 is modest and larger prospective studies in the future might be able to corroborate these findings with more certainty. Only 14 patients received a bone graft. This is a small number, however, the negative impact of a synthetic bone graft on the time to union and deep tissue infection rates seems to be a dominant theme when reviewing our findings. Given the novel nature of these results, with potentially serious ramifications for clinical practice in future, we believe that these results are relevant for all orthopedic surgeons managing tibial fractures in the context of delayed union and infection.

In conclusion, tibial fractures are significant injuries, which are highly demanding on the treating surgeon, hospital resources, and the patients' physical and psychological well-being. These cases often require multiple procedures and multiple admissions to the hospital before union is achieved. The majority of patients will experience a delayed union and the external fixator will be applied for a significant period of time, often leaving residual physical limitations.

Time to union is negatively impacted by the use of bone graft. With the subgroup analysis, it appears that the use of any form of synthetic bone graft will lengthen the time to union when used in combination with autograft, allograft, or when used alone. Best times to union are achieved when autograft is used alone without any other combination of bone graft type. The use of a synthetic bone graft also significantly increases the rate of deep tissue infection in this cohort, a complication often requiring further surgical intervention and considerable patient morbidity.

## Conclusions

Within the limitations of this pilot study, we conclude that the novel findings are sufficiently important to recommend the use of autograft alone when treating bone defects in tibial fractures with external fixators. The relationship between rhBMP-7, time to union, and deep tissue infection need to be further investigated with large-scale, prospective, randomized control trials in the future.
